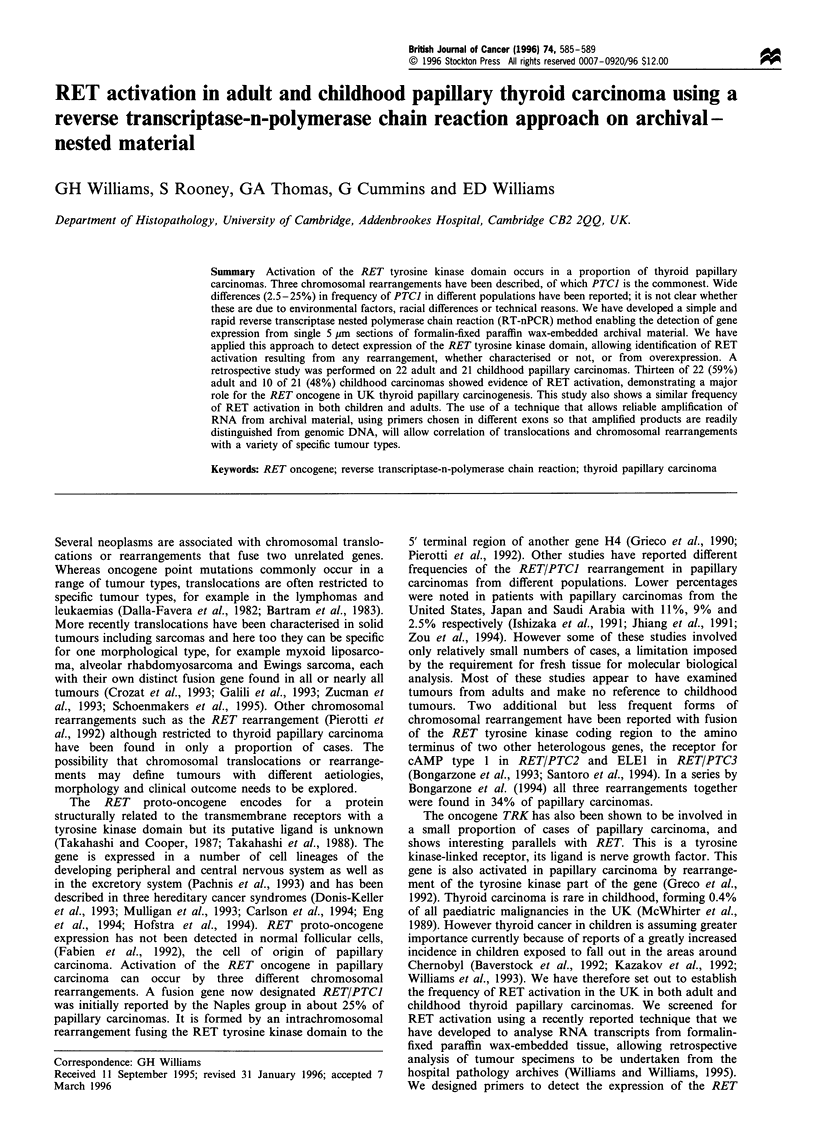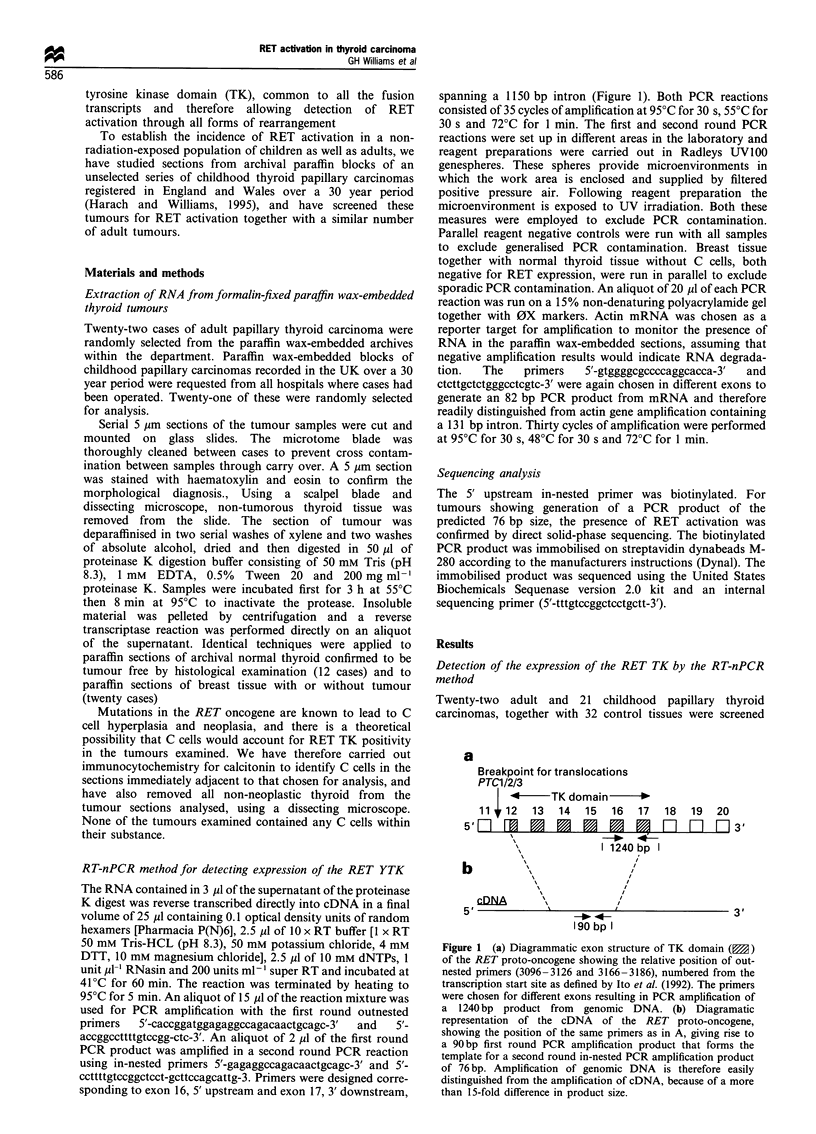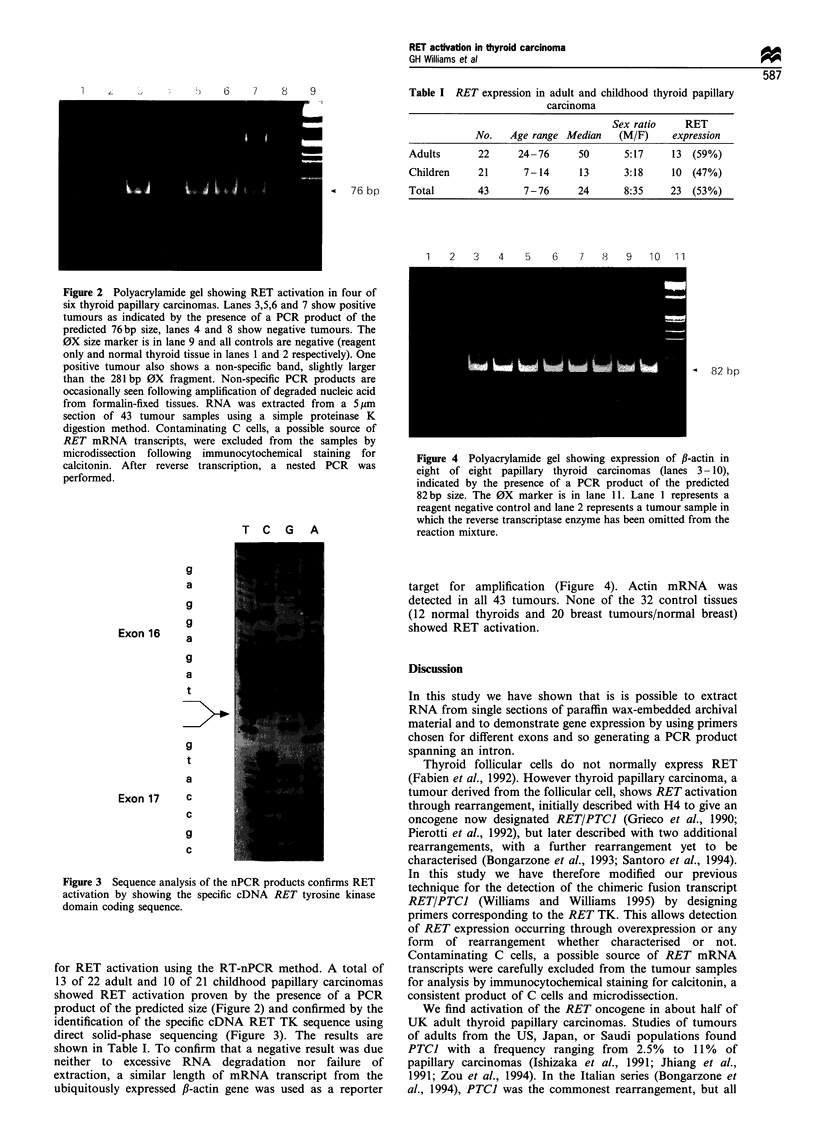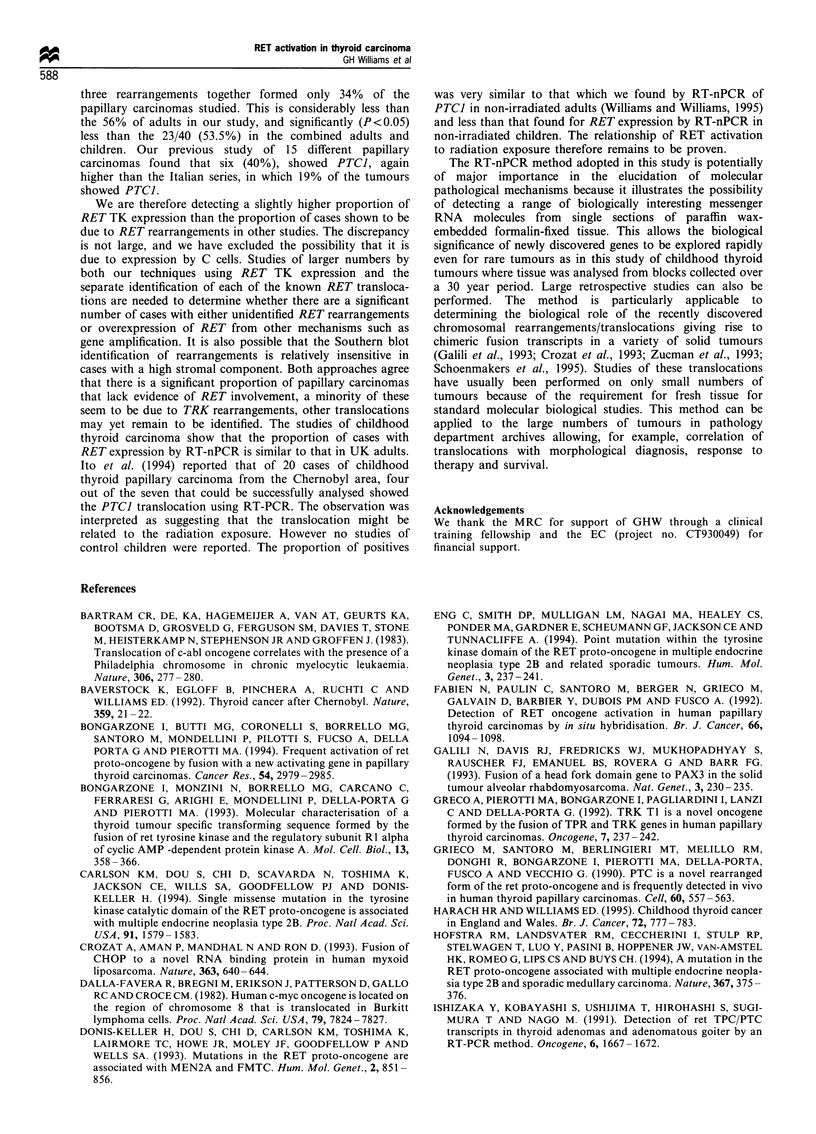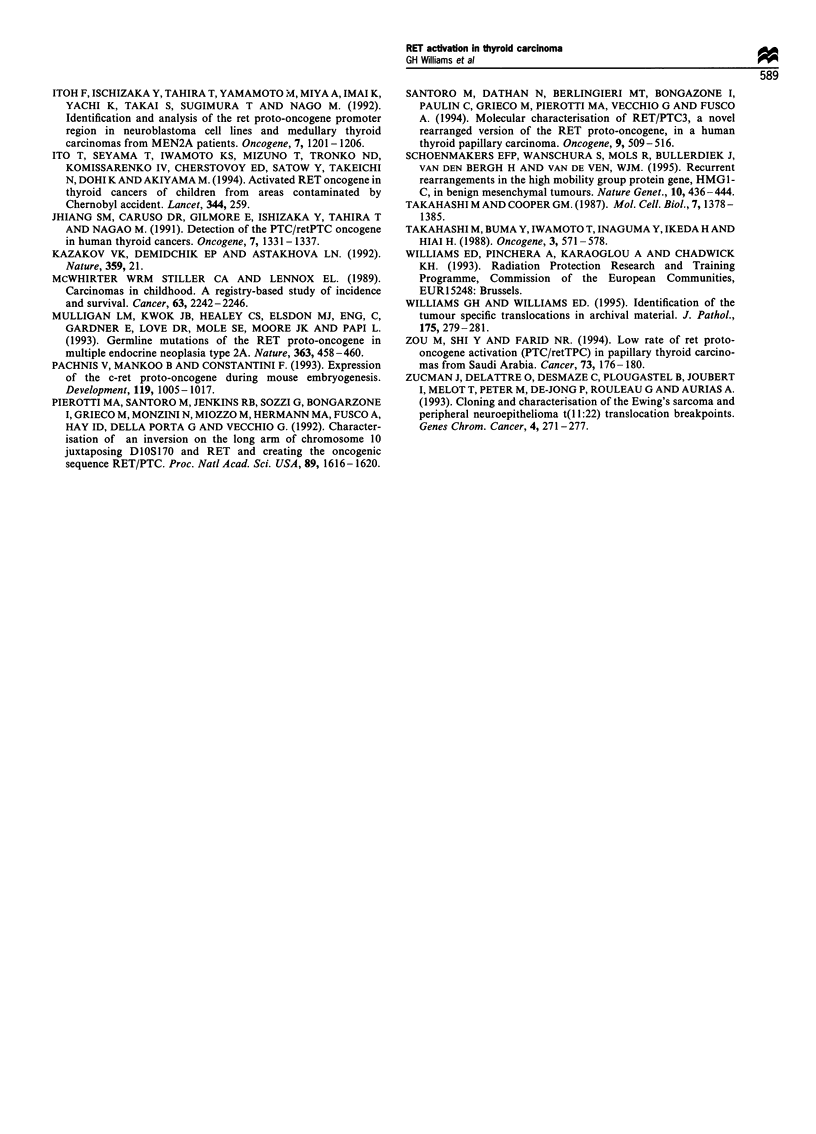# RET activation in adult and childhood papillary thyroid carcinoma using a reverse transcriptase-n-polymerase chain reaction approach on archival-nested material.

**DOI:** 10.1038/bjc.1996.405

**Published:** 1996-08

**Authors:** G. H. Williams, S. Rooney, G. A. Thomas, G. Cummins, E. D. Williams

**Affiliations:** Department of Histopathology, University of Cambridge, Addenbrookes Hospital, UK.

## Abstract

**Images:**


British Journal of Cancer (1996) 74, 585-589
© 1996 Stockton Press All rights reserved 0007-0920/96 $12.00

### RET activation in adult and childhood papillary thyroid carcinoma using a reverse transcriptase-n-polymerase chain reaction approach on archival-nested material

GH Williams, S Rooney, GA Thomas, G Cummins and ED Williams

*Department of Histopathology, University of Cambridge, Addenbrookes Hospital, Cambridge CB2 2QQ, UK.*

**Summary** Activation of the *RET* tyrosine kinase domain occurs in a proportion of thyroid papillary carcinomas. Three chromosomal rearrangements have been described, of which *PTC1* is the commonest. Wide differences (2.5-25%) in frequency of *PTC1* in different populations have been reported; it is not clear whether these are due to environmental factors, racial differences or technical reasons. We have developed a simple and rapid reverse transcriptase nested polymerase chain reaction (RT-nPCR) method enabling the detection of gene expression from single 5 μm sections of formalin-fixed paraffin wax-embedded archival material. We have applied this approach to detect expression of the *RET* tyrosine kinase domain, allowing identification of RET activation resulting from any rearrangement, whether characterised or not, or from overexpression. A retrospective study was performed on 22 adult and 21 childhood papillary carcinomas. Thirteen of 22 (59%) adult and 10 of 21 (48%) childhood carcinomas showed evidence of RET activation, demonstrating a major role for the *RET* oncogene in UK thyroid papillary carcinogenesis. This study also shows a similar frequency of RET activation in both children and adults. The use of a technique that allows reliable amplification of RNA from archival material, using primers chosen in different exons so that amplified products are readily distinguished from genomic DNA, will allow correlation of translocations and chromosomal rearrangements with a variety of specific tumour types.

**Keywords:** *RET* oncogene; reverse transcriptase-n-polymerase chain reaction; thyroid papillary carcinoma

Several neoplasms are associated with chromosomal translocations or rearrangements that fuse two unrelated genes. Whereas oncogene point mutations commonly occur in a range of tumour types, translocations are often restricted to specific tumour types, for example in the lymphomas and leukaemias (Dalla-Favera *et al.*, 1982; Bartram *et al.*, 1983). More recently translocations have been characterised in solid tumours including sarcomas and here too they can be specific for one morphological type, for example myxoid liposarcoma, alveolar rhabdomyosarcoma and Ewings sarcoma, each with their own distinct fusion gene found in all or nearly all tumours (Crozat *et al.*, 1993; Galili *et al.*, 1993; Zucman *et al.*, 1993; Schoenmakers *et al.*, 1995). Other chromosomal rearrangements such as the *RET* rearrangement (Pierotti *et al.*, 1992) although restricted to thyroid papillary carcinoma have been found in only a proportion of cases. The possibility that chromosomal translocations or rearrangements may define tumours with different aetiologies, morphology and clinical outcome needs to be explored.

The *RET* proto-oncogene encodes for a protein structurally related to the transmembrane receptors with a tyrosine kinase domain but its putative ligand is unknown (Takahashi and Cooper, 1987; Takahashi *et al.*, 1988). The gene is expressed in a number of cell lineages of the developing peripheral and central nervous system as well as in the excretory system (Pachnis *et al.*, 1993) and has been described in three hereditary cancer syndromes (Donis-Keller *et al.*, 1993; Mulligan *et al.*, 1993; Carlson *et al.*, 1994; Eng *et al.*, 1994; Hofstra *et al.*, 1994). *RET* proto-oncogene expression has not been detected in normal follicular cells, (Fabien *et al.*, 1992), the cell of origin of papillary carcinoma. Activation of the *RET* oncogene in papillary carcinoma can occur by three different chromosomal rearrangements. A fusion gene now designated *RET/PTC1* was initially reported by the Naples group in about 25% of papillary carcinomas. It is formed by an intrachromosomal rearrangement fusing the RET tyrosine kinase domain to the 5′ terminal region of another gene H4 (Grieco *et al.*, 1990; Pierotti *et al.*, 1992). Other studies have reported different frequencies of the *RET/PTC1* rearrangement in papillary carcinomas from different populations. Lower percentages were noted in patients with papillary carcinomas from the United States, Japan and Saudi Arabia with 11%, 9% and 2.5% respectively (Ishizaka *et al.*, 1991; Jhiang *et al.*, 1991; Zou *et al.*, 1994). However some of these studies involved only relatively small numbers of cases, a limitation imposed by the requirement for fresh tissue for molecular biological analysis. Most of these studies appear to have examined tumours from adults and make no reference to childhood tumours. Two additional but less frequent forms of chromosomal rearrangement have been reported with fusion of the *RET* tyrosine kinase coding region to the amino terminus of two other heterologous genes, the receptor for cAMP type 1 in *RET/PTC2* and ELE1 in *RET/PTC3* (Bongarzone *et al.*, 1993; Santoro *et al.*, 1994). In a series by Bongarzone *et al.* (1994) all three rearrangements together were found in 34% of papillary carcinomas.

The oncogene *TRK* has also been shown to be involved in a small proportion of cases of papillary carcinoma, and shows interesting parallels with *RET*. This is a tyrosine kinase-linked receptor, its ligand is nerve growth factor. This gene is also activated in papillary carcinoma by rearrangement of the tyrosine kinase part of the gene (Greco *et al.*, 1992). Thyroid carcinoma is rare in childhood, forming 0.4% of all paediatric malignancies in the UK (McWhirter *et al.*, 1989). However thyroid cancer in children is assuming greater importance currently because of reports of a greatly increased incidence in children exposed to fall out in the areas around Chernobyl (Baverstock *et al.*, 1992; Kazakov *et al.*, 1992; Williams *et al.*, 1993). We have therefore set out to establish the frequency of RET activation in the UK in both adult and childhood thyroid papillary carcinomas. We screened for RET activation using a recently reported technique that we have developed to analyse RNA transcripts from formalin-fixed paraffin wax-embedded tissue, allowing retrospective analysis of tumour specimens to be undertaken from the hospital pathology archives (Williams and Williams, 1995). We designed primers to detect the expression of the *RET*

Correspondence: GH Williams
Received 11 September 1995; revised 31 January 1996; accepted 7 March 1996

tyrosine kinase domain (TK), common to all the fusion transcripts and therefore allowing detection of RET activation through all forms of rearrangement

To establish the incidence of RET activation in a non-radiation-exposed population of children as well as adults, we have studied sections from archival paraffin blocks of an unselected series of childhood thyroid papillary carcinomas registered in England and Wales over a 30 year period (Harach and Williams, 1995), and have screened these tumours for RET activation together with a similar number of adult tumours.

### Materials and methods

*Extraction of RNA from formalin-fixed paraffin wax-embedded thyroid tumours*

Twenty-two cases of adult papillary thyroid carcinoma were randomly selected from the paraffin wax-embedded archives within the department. Paraffin wax-embedded blocks of childhood papillary carcinomas recorded in the UK over a 30 year period were requested from all hospitals where cases had been operated. Twenty-one of these were randomly selected for analysis.

Serial 5 $\mu$m sections of the tumour samples were cut and mounted on glass slides. The microtome blade was thoroughly cleaned between cases to prevent cross contamination between samples through carry over. A 5 $\mu$m section was stained with haematoxylin and eosin to confirm the morphological diagnosis., Using a scalpel blade and dissecting microscope, non-tumorous thyroid tissue was removed from the slide. The section of tumour was deparaffinised in two serial washes of xylene and two washes of absolute alcohol, dried and then digested in 50 $\mu$l of proteinase K digestion buffer consisting of 50 mM Tris (pH 8.3), 1 mM EDTA, 0.5% Tween 20 and 200 mg ml$^{-1}$ proteinase K. Samples were incubated first for 3 h at 55°C then 8 min at 95°C to inactivate the protease. Insoluble material was pelleted by centrifugation and a reverse transcriptase reaction was performed directly on an aliquot of the supernatant. Identical techniques were applied to paraffin sections of archival normal thyroid confirmed to be tumour free by histological examination (12 cases) and to paraffin sections of breast tissue with or without tumour (twenty cases)

Mutations in the *RET* oncogene are known to lead to C cell hyperplasia and neoplasia, and there is a theoretical possibility that C cells would account for RET TK positivity in the tumours examined. We have therefore carried out immunocytochemistry for calcitonin to identify C cells in the sections immediately adjacent to that chosen for analysis, and have also removed all non-neoplastic thyroid from the tumour sections analysed, using a dissecting microscope. None of the tumours examined contained any C cells within their substance.

*RT-nPCR method for detecting expression of the RET YTK*

The RNA contained in 3 $\mu$l of the supernatant of the proteinase K digest was reverse transcribed directly into cDNA in a final volume of 25 $\mu$l containing 0.1 optical density units of random hexamers [Pharmacia P(N)6], 2.5 $\mu$l of 10 × RT buffer [1 × RT 50 mM Tris-HCL (pH 8.3), 50 mM potassium chloride, 4 mM DTT, 10 mM magnesium chloride], 2.5 $\mu$l of 10 mM dNTPs, 1 unit $\mu$l$^{-1}$ RNasin and 200 units ml$^{-1}$ super RT and incubated at 41°C for 60 min. The reaction was terminated by heating to 95°C for 5 min. An aliquot of 15 $\mu$l of the reaction mixture was used for PCR amplification with the first round outnested primers 5′-caccggatggagaggccagacaactgcagc-3′ and 5′-accggcctttgtccgg-ctc-3′. An aliquot of 2 $\mu$l of the first round PCR product was amplified in a second round PCR reaction using in-nested primers 5′-gagaggccagacaactgcagc-3′ and 5′-ccttttgtccggctcct-gcttccagcattg-3. Primers were designed corresponding to exon 16, 5′ upstream and exon 17, 3′ downstream, spanning a 1150 bp intron (Figure 1). Both PCR reactions consisted of 35 cycles of amplification at 95°C for 30 s, 55°C for 30 s and 72°C for 1 min. The first and second round PCR reactions were set up in different areas in the laboratory and reagent preparations were carried out in Radleys UV100 genespheres. These spheres provide microenvironments in which the work area is enclosed and supplied by filtered positive pressure air. Following reagent preparation the microenvironment is exposed to UV irradiation. Both these measures were employed to exclude PCR contamination. Parallel reagent negative controls were run with all samples to exclude generalised PCR contamination. Breast tissue together with normal thyroid tissue without C cells, both negative for RET expression, were run in parallel to exclude sporadic PCR contamination. An aliquot of 20 $\mu$l of each PCR reaction was run on a 15% non-denaturing polyacrylamide gel together with ØX markers. Actin mRNA was chosen as a reporter target for amplification to monitor the presence of RNA in the paraffin wax-embedded sections, assuming that negative amplification results would indicate RNA degradation. The primers 5′-gtggggcgccccaggcacca-3′ and ctcttgctctgggcctcgtc-3′ were again chosen in different exons to generate an 82 bp PCR product from mRNA and therefore readily distinguished from actin gene amplification containing a 131 bp intron. Thirty cycles of amplification were performed at 95°C for 30 s, 48°C for 30 s and 72°C for 1 min.

*Sequencing analysis*

The 5′ upstream in-nested primer was biotinylated. For tumours showing generation of a PCR product of the predicted 76 bp size, the presence of RET activation was confirmed by direct solid-phase sequencing. The biotinylated PCR product was immobilised on streptavidin dynabeads M-280 according to the manufacturers instructions (Dynal). The immobilised product was sequenced using the United States Biochemicals Sequenase version 2.0 kit and an internal sequencing primer (5′-tttgtccggctcctgctt-3′).

### Results

*Detection of the expression of the RET TK by the RT-nPCR method*

Twenty-two adult and 21 childhood papillary thyroid carcinomas, together with 32 control tissues were screened

**Figure 1** (**a**) Diagrammatic exon structure of TK domain (▨) of the *RET* proto-oncogene showing the relative position of out-nested primers (3096–3126 and 3166–3186), numbered from the transcription start site as defined by Ito *et al.* (1992). The primers were chosen for different exons resulting in PCR amplification of a 1240 bp product from genomic DNA. (**b**) Diagramatic representation of the cDNA of the *RET* proto-oncogene, showing the position of the same primers as in A, giving rise to a 90 bp first round PCR amplification product that forms the template for a second round in-nested PCR amplification product of 76 bp. Amplification of genomic DNA is therefore easily distinguished from the amplification of cDNA, because of a more than 15-fold difference in product size.

**Figure 2** Polyacrylamide gel showing RET activation in four of six thyroid papillary carcinomas. Lanes 3,5,6 and 7 show positive tumours as indicated by the presence of a PCR product of the predicted 76 bp size, lanes 4 and 8 show negative tumours. The ØX size marker is in lane 9 and all controls are negative (reagent only and normal thyroid tissue in lanes 1 and 2 respectively). One positive tumour also shows a non-specific band, slightly larger than the 281 bp ØX fragment. Non-specific PCR products are occasionally seen following amplification of degraded nucleic acid from formalin-fixed tissues. RNA was extracted from a 5 $\mu$m section of 43 tumour samples using a simple proteinase K digestion method. Contaminating C cells, a possible source of *RET* mRNA transcripts, were excluded from the samples by microdissection following immunocytochemical staining for calcitonin. After reverse transcription, a nested PCR was performed.

**Figure 3** Sequence analysis of the nPCR products confirms RET activation by showing the specific cDNA *RET* tyrosine kinase domain coding sequence.

for RET activation using the RT-nPCR method. A total of 13 of 22 adult and 10 of 21 childhood papillary carcinomas showed RET activation proven by the presence of a PCR product of the predicted size (Figure 2) and confirmed by the identification of the specific cDNA RET TK sequence using direct solid-phase sequencing (Figure 3). The results are shown in Table I. To confirm that a negative result was due neither to excessive RNA degradation nor failure of extraction, a similar length of mRNA transcript from the ubiquitously expressed $\beta$-actin gene was used as a reporter target for amplification (Figure 4). Actin mRNA was detected in all 43 tumours. None of the 32 control tissues (12 normal thyroids and 20 breast tumours/normal breast) showed RET activation.

**Table I** *RET* expression in adult and childhood thyroid papillary carcinoma

| | *No.* | *Age range* | *Median* | *Sex ratio* (M/F) | RET *expression* |
|---|---|---|---|---|---|
| Adults | 22 | 24–76 | 50 | 5:17 | 13 (59%) |
| Children | 21 | 7–14 | 13 | 3:18 | 10 (47%) |
| Total | 43 | 7–76 | 24 | 8:35 | 23 (53%) |

**Figure 4** Polyacrylamide gel showing expression of $\beta$-actin in eight of eight papillary thyroid carcinomas (lanes 3–10), indicated by the presence of a PCR product of the predicted 82 bp size. The ØX marker is in lane 11. Lane 1 represents a reagent negative control and lane 2 represents a tumour sample in which the reverse transcriptase enzyme has been omitted from the reaction mixture.

### Discussion

In this study we have shown that is is possible to extract RNA from single sections of paraffin wax-embedded archival material and to demonstrate gene expression by using primers chosen for different exons and so generating a PCR product spanning an intron.

Thyroid follicular cells do not normally express RET (Fabien *et al.*, 1992). However thyroid papillary carcinoma, a tumour derived from the follicular cell, shows *RET* activation through rearrangement, initially described with H4 to give an oncogene now designated *RET/PTC1* (Grieco *et al.*, 1990; Pierotti *et al.*, 1992), but later described with two additional rearrangements, with a further rearrangement yet to be characterised (Bongarzone *et al.*, 1993; Santoro *et al.*, 1994). In this study we have therefore modified our previous technique for the detection of the chimeric fusion transcript *RET/PTC1* (Williams and Williams 1995) by designing primers corresponding to the *RET* TK. This allows detection of *RET* expression occurring through overexpression or any form of rearrangement whether characterised or not. Contaminating C cells, a possible source of *RET* mRNA transcripts were carefully excluded from the tumour samples for analysis by immunocytochemical staining for calcitonin, a consistent product of C cells and microdissection.

We find activation of the *RET* oncogene in about half of UK adult thyroid papillary carcinomas. Studies of tumours of adults from the US, Japan, or Saudi populations found *PTC1* with a frequency ranging from 2.5% to 11% of papillary carcinomas (Ishizaka *et al.*, 1991; Jhiang *et al.*, 1991; Zou *et al.*, 1994). In the Italian series (Bongarzone *et al.*, 1994), *PTC1* was the commonest rearrangement, but all three rearrangements together formed only 34% of the papillary carcinomas studied. This is considerably less than the 56% of adults in our study, and significantly ($P<0.05$) less than the 23/40 (53.5%) in the combined adults and children. Our previous study of 15 different papillary carcinomas found that six (40%), showed *PTC1*, again higher than the Italian series, in which 19% of the tumours showed *PTC1*.

We are therefore detecting a slightly higher proportion of *RET* TK expression than the proportion of cases shown to be due to *RET* rearrangements in other studies. The discrepancy is not large, and we have excluded the possibility that it is due to expression by C cells. Studies of larger numbers by both our techniques using *RET* TK expression and the separate identification of each of the known *RET* translocations are needed to determine whether there are a significant number of cases with either unidentified *RET* rearrangements or overexpression of *RET* from other mechanisms such as gene amplification. It is also possible that the Southern blot identification of rearrangements is relatively insensitive in cases with a high stromal component. Both approaches agree that there is a significant proportion of papillary carcinomas that lack evidence of *RET* involvement, a minority of these seem to be due to *TRK* rearrangements, other translocations may yet remain to be identified. The studies of childhood thyroid carcinoma show that the proportion of cases with *RET* expression by RT-nPCR is similar to that in UK adults. Ito *et al.* (1994) reported that of 20 cases of childhood thyroid papillary carcinoma from the Chernobyl area, four out of the seven that could be successfully analysed showed the *PTC1* translocation using RT-PCR. The observation was interpreted as suggesting that the translocation might be related to the radiation exposure. However no studies of control children were reported. The proportion of positives was very similar to that which we found by RT-nPCR of *PTC1* in non-irradiated adults (Williams and Williams, 1995) and less than that found for *RET* expression by RT-nPCR in non-irradiated children. The relationship of RET activation to radiation exposure therefore remains to be proven.

The RT-nPCR method adopted in this study is potentially of major importance in the elucidation of molecular pathological mechanisms because it illustrates the possibility of detecting a range of biologically interesting messenger RNA molecules from single sections of paraffin wax-embedded formalin-fixed tissue. This allows the biological significance of newly discovered genes to be explored rapidly even for rare tumours as in this study of childhood thyroid tumours where tissue was analysed from blocks collected over a 30 year period. Large retrospective studies can also be performed. The method is particularly applicable to determining the biological role of the recently discovered chromosomal rearrangements/translocations giving rise to chimeric fusion transcripts in a variety of solid tumours (Galili *et al.*, 1993; Crozat *et al.*, 1993; Zucman *et al.*, 1993; Schoenmakers *et al.*, 1995). Studies of these translocations have usually been performed on only small numbers of tumours because of the requirement for fresh tissue for standard molecular biological studies. This method can be applied to the large numbers of tumours in pathology department archives allowing, for example, correlation of translocations with morphological diagnosis, response to therapy and survival.

**Acknowledgements**
We thank the MRC for support of GHW through a clinical training fellowship and the EC (project no. CT930049) for financial support.

### References

BARTRAM CR, DE, KA, HAGEMEIJER A, VAN AT, GEURTS KA, BOOTSMA D, GROSVELD G, FERGUSON SM, DAVIES T, STONE M, HEISTERKAMP N, STEPHENSON JR AND GROFFEN J. (1983). Translocation of c-abl oncogene correlates with the presence of a Philadelphia chromosome in chronic myelocytic leukaemia. *Nature*, **306**, 277–280.

BAVERSTOCK K, EGLOFF B, PINCHERA A, RUCHTI C AND WILLIAMS ED. (1992). Thyroid cancer after Chernobyl. *Nature*, **359**, 21–22.

BONGARZONE I, BUTTI MG, CORONELLI S, BORRELLO MG, SANTORO M, MONDELLINI P, PILOTTI S, FUCSO A, DELLA PORTA G AND PIEROTTI MA. (1994). Frequent activation of ret proto-oncogene by fusion with a new activating gene in papillary thyroid carcinomas. *Cancer Res.*, **54**, 2979–2985.

BONGARZONE I, MONZINI N, BORRELLO MG, CARCANO C, FERRARESI G, ARIGHI E, MONDELLINI P, DELLA-PORTA G AND PIEROTTI MA. (1993). Molecular characterisation of a thyroid tumour specific transforming sequence formed by the fusion of ret tyrosine kinase and the regulatory subunit R1 alpha of cyclic AMP -dependent protein kinase A. *Mol. Cell. Biol.*, **13**, 358–366.

CARLSON KM, DOU S, CHI D, SCAVARDA N, TOSHIMA K, JACKSON CE, WILLS SA, GOODFELLOW PJ AND DONIS-KELLER H. (1994). Single missense mutation in the tyrosine kinase catalytic domain of the RET proto-oncogene is associated with multiple endocrine neoplasia type 2B. *Proc. Natl Acad. Sci. USA*, **91**, 1579–1583.

CROZAT A, AMAN P, MANDHAL N AND RON D. (1993). Fusion of CHOP to a novel RNA binding protein in human myxoid liposarcoma. *Nature*, **363**, 640–644.

DALLA-FAVERA R, BREGNI M, ERIKSON J, PATTERSON D, GALLO RC AND CROCE CM. (1982). Human c-myc oncogene is located on the region of chromosome 8 that is translocated in Burkitt lymphoma cells. *Proc. Natl Acad. Sci. USA*, **79**, 7824–7827.

DONIS-KELLER H, DOU S, CHI D, CARLSON KM, TOSHIMA K, LAIRMORE TC, HOWE JR, MOLEY JF, GOODFELLOW P AND WELLS SA. (1993). Mutations in the RET proto-oncogene are associated with MEN2A and FMTC. *Hum. Mol. Genet.*, **2**, 851–856.

ENG C, SMITH DP, MULLIGAN LM, NAGAI MA, HEALEY CS, PONDER MA, GARDNER E, SCHEUMANN GF, JACKSON CE AND TUNNACLIFFE A. (1994). Point mutation within the tyrosine kinase domain of the RET proto-oncogene in multiple endocrine neoplasia type 2B and related sporadic tumours. *Hum. Mol. Genet.*, **3**, 237–241.

FABIEN N, PAULIN C, SANTORO M, BERGER N, GRIECO M, GALVAIN D, BARBIER Y, DUBOIS PM AND FUSCO A. (1992). Detection of RET oncogene activation in human papillary thyroid carcinomas by *in situ* hybridisation. *Br. J. Cancer*, **66**, 1094–1098.

GALILI N, DAVIS RJ, FREDRICKS WJ, MUKHOPADHYAY S, RAUSCHER FJ, EMANUEL BS, ROVERA G AND BARR FG. (1993). Fusion of a head fork domain gene to PAX3 in the solid tumour alveolar rhabdomyosarcoma. *Nat. Genet.*, **3**, 230–235.

GRECO A, PIEROTTI MA, BONGARZONE I, PAGLIARDINI I, LANZI C AND DELLA-PORTA G. (1992). TRK T1 is a novel oncogene formed by the fusion of TPR and TRK genes in human papillary thyroid carcinomas. *Oncogene*, **7**, 237–242.

GRIECO M, SANTORO M, BERLINGIERI MT, MELILLO RM, DONGHI R, BONGARZONE I, PIEROTTI MA, DELLA-PORTA, FUSCO A AND VECCHIO G. (1990). PTC is a novel rearranged form of the ret proto-oncogene and is frequently detected in vivo in human thyroid papillary carcinomas. *Cell*, **60**, 557–563.

HARACH HR AND WILLIAMS ED. (1995). Childhood thyroid cancer in England and Wales. *Br. J. Cancer*, **72**, 777–783.

HOFSTRA RM, LANDSVATER RM, CECCHERINI I, STULP RP, STELWAGEN T, LUO Y, PASINI B, HOPPENER JW, VAN-AMSTEL HK, ROMEO G, LIPS CS AND BUYS CH. (1994), A mutation in the RET proto-oncogene associated with multiple endocrine neoplasia type 2B and sporadic medullary carcinoma. *Nature*, **367**, 375–376.

ISHIZAKA Y, KOBAYASHI S, USHIJIMA T, HIROHASHI S, SUGIMURA T AND NAGO M. (1991). Detection of ret TPC/PTC transcripts in thyroid adenomas and adenomatous goiter by an RT-PCR method. *Oncogene*, **6**, 1667–1672.

ITOH F, ISCHIZAKA Y, TAHIRA T, YAMAMOTO M, MIYA A, IMAI K, YACHI K, TAKAI S, SUGIMURA T AND NAGO M. (1992). Identification and analysis of the ret proto-oncogene promoter region in neuroblastoma cell lines and medullary thyroid carcinomas from MEN2A patients. *Oncogene*, **7**, 1201–1206.

ITO T, SEYAMA T, IWAMOTO KS, MIZUNO T, TRONKO ND, KOMISSARENKO IV, CHERSTOVOY ED, SATOW Y, TAKEICHI N, DOHI K AND AKIYAMA M. (1994). Activated RET oncogene in thyroid cancers of children from areas contaminated by Chernobyl accident. *Lancet*, **344**, 259.

JHIANG SM, CARUSO DR, GILMORE E, ISHIZAKA Y, TAHIRA T AND NAGAO M. (1991). Detection of the PTC/retPTC oncogene in human thyroid cancers. *Oncogene*, **7**, 1331–1337.

KAZAKOV VK, DEMIDCHIK EP AND ASTAKHOVA LN. (1992). *Nature*, **359**, 21.

MCWHIRTER WRM STILLER CA AND LENNOX EL. (1989). Carcinomas in childhood. A registry-based study of incidence and survival. *Cancer*, **63**, 2242–2246.

MULLIGAN LM, KWOK JB, HEALEY CS, ELSDON MJ, ENG, C, GARDNER E, LOVE DR, MOLE SE, MOORE JK AND PAPI L. (1993). Germline mutations of the RET proto-oncogene in multiple endocrine neoplasia type 2A. *Nature*, **363**, 458–460.

PACHNIS V, MANKOO B AND CONSTANTINI F. (1993). Expression of the c-ret proto-oncogene during mouse embryogenesis. *Development*, **119**, 1005–1017.

PIEROTTI MA, SANTORO M, JENKINS RB, SOZZI G, BONGARZONE I, GRIECO M, MONZINI N, MIOZZO M, HERMANN MA, FUSCO A, HAY ID, DELLA PORTA G AND VECCHIO G. (1992). Characterisation of an inversion on the long arm of chromosome 10 juxtaposing D10S170 and RET and creating the oncogenic sequence RET/PTC. *Proc. Natl Acad. Sci. USA*, **89**, 1616–1620.

SANTORO M, DATHAN N, BERLINGIERI MT, BONGAZONE I, PAULIN C, GRIECO M, PIEROTTI MA, VECCHIO G AND FUSCO A. (1994). Molecular characterisation of RET/PTC3, a novel rearranged version of the RET proto-oncogene, in a human thyroid papillary carcinoma. *Oncogene*, **9**, 509–516.

SCHOENMAKERS EFP, WANSCHURA S, MOLS R, BULLERDIEK J, VAN DEN BERGH H AND VAN DE VEN, WJM. (1995). Recurrent rearrangements in the high mobility group protein gene, HMG1-C, in benign mesenchymal tumours. *Nature Genet.*, **10**, 436–444.

TAKAHASHI M AND COOPER GM. (1987). *Mol. Cell. Biol.*, **7**, 1378–1385.

TAKAHASHI M, BUMA Y, IWAMOTO T, INAGUMA Y, IKEDA H AND HIAI H. (1988). *Oncogene*, **3**, 571–578.

WILLIAMS ED, PINCHERA A, KARAOGLOU A AND CHADWICK KH. (1993). Radiation Protection Research and Training Programme, Commission of the European Communities, EUR15248: Brussels.

WILLIAMS GH AND WILLIAMS ED. (1995). Identification of the tumour specific translocations in archival material. *J. Pathol.*, **175**, 279–281.

ZOU M, SHI Y AND FARID NR. (1994). Low rate of ret proto-oncogene activation (PTC/retTPC) in papillary thyroid carcinomas from Saudi Arabia. *Cancer*, **73**, 176–180.

ZUCMAN J, DELATTRE O, DESMAZE C, PLOUGASTEL B, JOUBERT I, MELOT T, PETER M, DE-JONG P, ROULEAU G AND AURIAS A. (1993). Cloning and characterisation of the Ewing's sarcoma and peripheral neuroepithelioma t(11:22) translocation breakpoints. *Genes Chrom. Cancer*, **4**, 271–277.